# Structure-activity relationship of volatile compounds that induce defense-related genes in maize seedlings

**DOI:** 10.1080/15592324.2023.2234115

**Published:** 2023-07-16

**Authors:** Yasuhiro Tanaka, Kenya Fujita, Minori Date, Bunta Watanabe, Kenji Matsui

**Affiliations:** a Graduate School of Sciences and Technology for Innovation, Yamaguchi University, Yamaguchi, Japan; b Chemistry Laboratory, The Jikei University School of Medicine, Chofu, Japan

**Keywords:** Green leaf volatiles, *Zea mays*, plant communication, RT-qPCR, defense-gene induction

## Abstract

Volatile organic compounds mediate plant-to-plant communication, and plants receiving volatile cues can acquire greater defenses against attackers. It has been expected that volatiles are received by factors that eventually lead to the induction of defense-related gene expression; however, the nature of these factors remain unclear. Structure-activity relationship analysis of gene expression induction by volatiles should provide insights into the nature of these factors. We conducted a structure-activity relationship study using maize seedlings and (*Z*)-3-hexen-1-yl acetate (Z3HAC) as the lead compound. The acid portion of Z3HAC was not essential, and (*Z*)-3-hexen-1-ol (Z3HOL), which is formed after the hydrolysis of Z3HAC, is likely the structure essential for the upregulation of the genes. The double bond of Z3HOL is essential; however, its geometry is indistinguishable. Strict specificity was detected regarding the length of the methylene chain on the α- and ω-sides of the double bond, and therefore, the 3-hexen-1-ol structure was found to be the ultimate structure. This finding provides insight into the nature of the factors that interact with a volatile compound and subsequently activate signaling pathways, leading to the upregulation of a subset of defense genes.

## Introduction

When plants are attacked by herbivores or pathogens, they release a blend of volatile organic compounds. In general, volatiles emitted under such conditions can enhance plant fitness, as they repel attackers (direct defense) and, in some cases, attract their enemies (indirect defense).^1^ Furthermore, some plant volatiles induce the defense of neighboring plants surrounding the emitters.^2^ Although plant communication via volatile compounds was controversial, reliable studies had been conducted since the turn of the century, and a meta-analysis published in 2014^3^ indicated that volatile communication within and between plants is common. Now, many researchers are challenging to unravel the mechanism how plants perceive volatiles; however, it has not been completely elucidated.

Green leaf volatiles (GLVs) consist of six-carbon aliphatic aldehydes, alcohols, and esters. In intact plant tissues, the amounts of GLVs are usually low. However, when plant tissues are subjected to stimuli that cause cellular damage, they are instantly formed.^4^ Linolenic acid, either in its free or esterified form, is the most often used substrate for the biosynthesis of GLVs, and (*Z*)-3-hexenal (**3**, Z3HAL) is the first compound to be synthesized among GLVs. A portion of Z3HAL formed in damaged plant cells diffuses into the neighboring intact tissues, where it is reduced by cinnamaldehyde and hexenal reductase to (*Z*)-3-hexen-1-ol (**2**, Z3HOL).^5^ A portion of Z3HOL is further converted to (*Z*)-3-hexen-1-yl acetate (**1**, Z3HAC) by acetyltransferase.^6^ These series of reactions quickly occur, and in partially wounded Arabidopsis leaves, the peak emission of Z3HAL occurred at 30–45 s following damage, followed by Z3HOL at 2.5 min and Z3HAC at 4.5–5.5 min.^6^ Such rapid and transient GLV generation, known as GLV burst, contributes to the suppression of pathogens attempting to invade wounds and provides real-time information about the activity of herbivores toward predators.^7^ Real-time information is useful for predators searching for victims (herbivores) that vigorously bite plant leaves.^4^

GLVs are also involved in intra- and inter-plant interactions.^2^ The effects of GLVs on the induction of defense in receiving tissues or plants are found in phylogenetically distant plant species, such as maize, Arabidopsis, and tomato. Additionally, the mode of defense induced by each GLV compound appeared to differ. Therefore, plants perceive GLVs by multiple mechanisms. Among the examples of GLV-mediated plant-plant interactions reported thus far, the mechanism of the response of tomatoes to Z3HOL has been examined in detail, and it has been shown that Z3HOL is taken up by the receiver plant and converted into its glycoside, which causes insect feeding inhibition, thereby increasing the herbivore defense of the receiver plant.^8,9^ In this case, activation of the signal transduction pathway in the receiver tomato plant may not be essential, and glycosylation is thought to proceed via glycosyltransferases that were probably already present in the plant before treatment.^9^ On the other hand, in a variety of plants, GLV treatment directly induces expression of defense-related genes or stimulates the production of metabolites involved in defense. In some combinations of plants and GLVs, GLV treatment primes the receiver plant, and although GLV treatment alone does not show a marked difference in defense levels, pre-exposed plants show more rapid and greater defense than that of non-exposed plants when stresses, such as feeding damage, are applied after GLV treatment.^2^ These responses indicate that the signal transduction pathway involved in defense is activated in the receiver plants. In fact, signaling pathway components, such as mitogen-activated kinases and their interacting transcription factors (WRKYs), are activated by GLV treatment.^10^ GLVs also have the potential to modulate calcium influx in tomato leaves^11^ and calcium-related signaling in maize leaves.^12^ This means that GLVs are accepted by a factor in a plant and are converted into a signal that activates the defense system in the plant; however, this factor has not yet been identified.

**Figure 1.**
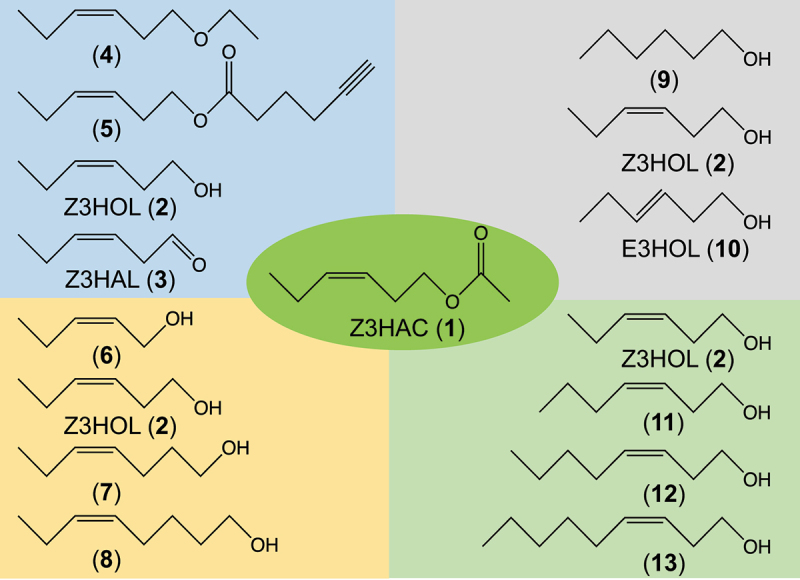
Compounds used in this study. (*Z*)-3-Hexen-1-yl acetate (**1**, Z3HAC), (*Z*)-3-hexen-1-ol (**2**, Z3HOL), (*Z*)-3-hexenal (**3**, Z3HAL), (*Z*)-3-hexen-1-yl ethyl ether (**4**), (*Z*)-3-hexen-1-yl 5-hexynoate (**5**), (*Z*)-2-penten-1-ol (**6**), (*Z*)-4-hepten-1-ol (**7**), (*Z*)-5-octen-1-ol (**8**), *n*-hexan-1-ol (**9**), (*E*)-3-hexen-1-ol (**10**), (*Z*)-3-hepten-1-ol (**11**), (*Z*)-3-octen-1-ol (**12**), and (*Z*)-3-nonen-1-ol (**13**).

One approach to identify this factor is to perform a structure-activity relationship analysis. By clarifying the key structure recognized by this factor, we can gain insight into its nature. Among GLVs, aldehydes such as (*E*)-2-hexenal (E2HAL) and Z3HAL react with nucleophilic biological substances, such as amines and thiols. E2HAL, in particular, has an unsaturated carbonyl structure that makes it susceptible to Michael addition reactions.^13,14^ It is expected that treatment with such compounds would alter the redox state in the plant by depleting intracellular thiols and amines, leading to the induction of defense-related plant responses. In this case, the compound that induces defense does not necessarily have to be a GLV but can be any aldehyde or unsaturated carbonyl that has some chemical reactivity.^14^ In fact, genes induced by E2HAL treatment were mostly related to response of plants against heat and drought stresses, with little induction of genes involved in defense against biotic stresses, and furthermore, the same abiotic stress response-related genes were also induced by diverse array of compounds harboring α,β-unsaturated carbonyl moiety other than E2HAL.^13^ In this context, it is unlikely that the defense is induced by the recognition of E2HAL, as a member of GLVs, by a specific factor. In addition, E2HAL and Z3HAL are not the major GLVs released by most plants under feeding damage.^15,16^ Accordingly, we decided that a structure-activity relationship analysis using E2HAL and Z3HAL as the lead compounds was not meaningful. We searched preceding papers reporting plant responses to Z3HAC and Z3HOL, which are generally released as the major constituents by feeding damage, and searched for a component of GLVs and a plant species in which direct gene induction can be clearly observed at relatively low concentrations of GLVs. Hu et al.^17^ reported that treating maize seedlings with relatively low concentrations of Z3HAC increased the expression of several genes potentially involved in herbivore defense, such as *ZmCyst*, *ZmAOS*, and *ZmTPS10*. Thus, we proceeded with a structure-activity relationship analysis based on this experimental system. Z3HAC was set as the lead compound, and thirteen compounds with different structures ranging from five to twelve carbons were used (Figure 1). Because the volatilities of the compounds used in this study vary extensively, controlling the gas concentration and gas-liquid partitioning of each compound is difficult. Therefore, we used an aqueous solution instead of vapor to treat the plants. It is also known that when plants are exposed to VOC vapors, some of the VOCs are adsorbed on the surface of the plant tissues.^2,15,18^ Therefore, it is likely that the response of plants to VOC treatment in solution at least partially mimics the response of plants to VOC vapor treatment.

## Materials and methods

### Plant materials

Maize (*Zea mays* L. cv. Canberra 90EX) (Takii, Kyoto, Japan) plants were grown in 6-cm-diameter pots using gardening soil (Takii) at 25°C under LED light (iD Series 40, Panasonic, Osaka, Japan) under a 12-h/12-h light/dark photoperiod with a light intensity of 137µmol m^−2^ s^−1^. Two-week-old plants were used in the present study.

### Synthesis of (*Z*)-3-hexen-1-yl ethyl ether (4) and (*Z*)-3-hexen-1-yl 5-hexynoate (5)

NMR spectra were obtained using a JEOL JNM-ECA600 spectrometer (600 MHz for ^1^H and 151 MHz for ^13^C). Chemical shifts were reported in parts per million relative to internal standards [tetramethylsilane (0.00 ppm) for ^1^H and CDCl_3_ (77.00 ppm) for ^13^C]. Reagents were used as received from commercial suppliers. Flash column chromatography on silica gel was performed using a Biotage Isolera One chromatograph with SNAP Ultra cartridges (silica gel, 25 µm).

Synthesis of **4**. A solution of **2** (5.20 g, 51.9 mmol) in anhydrous tetrahydrofuran (20 mL) was added dropwise to a mixture of NaH (60% in oil, 2.30 g, 57.5 mmol) and anhydrous tetrahydrofuran (50 mL) at 0°C. After 30 min of stirring at 0°C, a solution of EtI (9.50 g, 60.9 mmol) in anhydrous tetrahydrofuran (20 mL) was added dropwise to the mixture at 0°C, and stirring was continued for a further 17 h at room temperature. The reaction was quenched by the addition of crushed ice (50 g) at 0°C. The mixture was warmed to room temperature and the separated aqueous layer was extracted with Et_2_O (3 × 30 mL). The combined organic layers were washed with brine (50 mL), dried over anhydrous MgSO_4_, and filtered. The solvent was removed under normal pressure with heating (gradually warmed up to 80°C), and the residue was distilled under reduced pressure (108–111°C/37 kPa) to give a colorless oil (4.59 g). This product was revealed to be a mixture of desired **4** and unreacted **2** (ratio = 80/20, approximately) based on ^1^H NMR. The distillate was purified using flash column chromatography on silica gel (hexane/EtOAc = 90/10, v/v). The fraction containing **4** was collected, and the solvent was removed under normal pressure with heating (gradually warmed up to 100°C). The residue was further purified by distillation under reduced pressure to obtain **4** as a colorless oil (1.89 g, 28%). Bp 48–54°C/35 kPa [comparable to 66–68°C/50 mmHg.^19^]. ^1^H NMR (600 MHz, CDCl_3_, δ): 0.97 (3 H, t, *J* = 7.6 Hz), 1.20 (3 H, t, *J* = 6.9 Hz), 2.03–2.09 (2 H, m), 2.31–2.36 (2 H, m), 3.41 (2 H, t, *J* = 7.2 Hz), 3.49 (2 H, q, *J* = 6.9 Hz), 5.32–5.37 (1 H, m), 5.44–5.49 (1 H, m). ^13^C NMR (151 MHz, CDCl_3_, δ): 14.23, 15.16, 20.57, 27.84, 66.06, 70.26, 124.81, 133.57. The ^1^H and ^13^C NMR spectra of **5** were shown in Supplemental Figures S1 and S2, respectively.

Synthesis of **5**. A mixture of 5-hexynoic acid (510 mg, 4.55 mmol), **2** (513 mg, 5.12 mmol), 1-(3-dimethylaminopropyl)-3-ethylcarbodiimide hydrochloride (997 mg, 5.20 mmol), and anhydrous CH_2_Cl_2_ (4.0 mL) was successively treated with Et_3_N (0.80 mL, 5.7 mmol) and 4-dimethylaminopyridine (33.1 mg, 0.271 mmol) at 0°C. After stirring for 20 h at room temperature, the reaction mixture was subjected to flash column chromatography on silica gel (hexane/EtOAc = 95/5, v/v) to obtain **5** (760 mg, 86%) as a colorless oil. ^1^H NMR (600 MHz, CDCl_3_, δ): 0.97 (3 H, t, *J* = 7.6 Hz), 1.82–1.87 (2 H, m), 1.98 (1 H, t, *J* = 2.7 Hz), 2.03–2.09 (2 H, m), 2.26 (2 H, td, *J* = 6.9, 2.7 Hz), 2.36–2.40 (2 H, m), 2.45 (2 H, t, *J* = 7.6 Hz), 4.08 (2 H, t, *J* = 7.0 Hz), 5.29–5.34 (1 H, m), 5.48–5.33 (1 H, m). ^13^C NMR (151 MHz, CDCl_3_, δ): 14.15, 17.76, 20.51, 23.54, 26.64, 32.80, 63.84, 69.01, 83.19, 123.61, 134.49, 172.97. The ^1^H and ^13^C NMR spectra of **5** are shown in Supplementary Figures S3 and S4, respectively.

### Treatment of plants

Compounds used to treat maize seedlings other than **3**, **4**, and **5** were purchased from Fujifilm Wako Pure Chemicals (Osaka, Japan). (*Z*)-3-Hexenal (**3**, Z3HAL) was a generous gift from Zeon (Tokyo, Japan). For chemical treatment, a 1 mM solution of each compound was prepared with 0.1% (w/v) Tween 20 (Fujifilm Wako Pure Chemicals). Two milliliters of the solution were uniformly sprayed on four maize seedlings, and the treated plants were left for 2 h under light (40 µmol m^−2^ s^−1^). Control plants were sprayed with a 0.1% Tween 20 solution. Maize seedlings were essentially exposed to volatiles as reported by Hu et al.^17^ (Supplemental Figure S5). In brief, dispensers consisted of 4-mL glass vials (15 i.d. ×45 mm, Merck, Rahway, NJ) containing 0.2 mL of Z3HOL (**2**), or 8% (v/v) Z3HAC (**1**) dissolved in triethyl citrate were placed in 1-L glass separable flask. The dispensers were closed with the screw caps that contained a polytetrafluoroethylene (PTFE) septum, which was pierced with a 1-µL micropette (Hirschmann Minicaps, Merck). The separable flask with the dispensers was connected to 12-L polyethylene terephthalate (PET) bottles with PTFE tubing. Maize seedlings were placed in PET bottles. Compressed air generated using an oil-free air compressor (0.2 OP-5S, Hitachi Industrial Equipment Systems, Tokyo, Japan) was purified through a charcoal cartridge and introduced into a separable flask at a flow rate of 0.3 L min^−1^. Air-containing vaporized GLVs was introduced into the PET bottle. The air was taken from the top of the PET bottle at a flow rate of 0.2 L min^−1^ with a diaphragm pump (DAP-6D, Ulvac, Kanagawa, Japan). Surplus air was released through the openings of the PET bottle. The setup was placed in a room with sufficient ventilation at 25°C under light. The airflow was adjusted using a flow meter (RK20T; Kofloc, Kyoto, Japan). The control plants were treated in the same manner but without microdispensers.

### Gene expression analysis

Based on the results of a previous study.^17^, we selected *ZmCyst*, *ZmAOS*, and *ZmTPS10* as the genes to be monitored. After the compound treatment, the tips (5 cm from the top) of the primary leaves of maize seedlings were collected and RNA was extracted using a FavorPrep Plant Total RNA Purification Mini Kit (Favorgen, Ping Tung, Taiwan). After removing the DNA with a DNA-free DNA Removal Kit (Thermo Fisher Scientific, Waltham, MA), a portion of RNA (1 µg) was used to synthesize cDNA using ReverTra Ace qPCR Master Mix (Toyobo, Osaka, Japan). Quantitative reverse transcription PCR (RT-qPCR) was performed using a StePOnePlus System (Thermo Fisher Scientific) with SYBR Green Master Mix (Thermo Fisher Scientific). Primers and gene IDs are listed in Supplemental Table S1. The maize adenine phosphoribosyl transferase (*ZmAPT1*) gene was used as an internal standard to normalize cDNA concentration, and the relative expression levels of the target genes were obtained.

### Volatile analyses

Tenax TA 60/80 tube (GL Science, Tokyo, Japan) was conditioned at 250°C for 20 min with 60 mL min^−1^ nitrogen gas flow prior to use and set at the outlet of the PET bottle of the above setup made without maize seedlings. In the separable flask, the microdispenser containing 0.2 mL of Z3HOL (**2**) or 8% Z3HAC (**1**) in triethyl citrate was placed in separate flasks. The air was introduced into the separable flask at 0.3 L min^−1^ and withdrawn through the Tenax tube at 0.2 L min^−1^ for 1 hour. The volatiles collected in the tubes were analyzed using a thermal desorption GC/MS system (TQ8040NX equipped with TD-30 R, Shimadzu, Kyoto, Japan). Thermal desorption was carried out at 230°C for 10 min at 50 mL min^−1^ of nitrogen gas flow, and the volatiles were cryofocused onto the trap tube at −10°C. The trap-desorption was performed at 280°C for 5 min. Separation of the volatiles was carried out using a DB-WAX column (30 m × 0.25 mm, 0.25 µm thickness, Agilent Technologies, Santa Clara, CA). The column oven was set at 40°C for 5 min, programmed at a rate of 5°C min^−1^ to 200°C, and maintained at 200°C for 3 min. Carrier gas (He) was supplied at a constant pressure of 86.1 kPa. The mass detector was operated in the electron impact mode with an ionization energy of 70 eV. Z3HOL (**2**) and Z3HAC (**1**) were assigned by comparing the MS profiles and retention times of authentic compounds.

## Results

**Figure 2.**
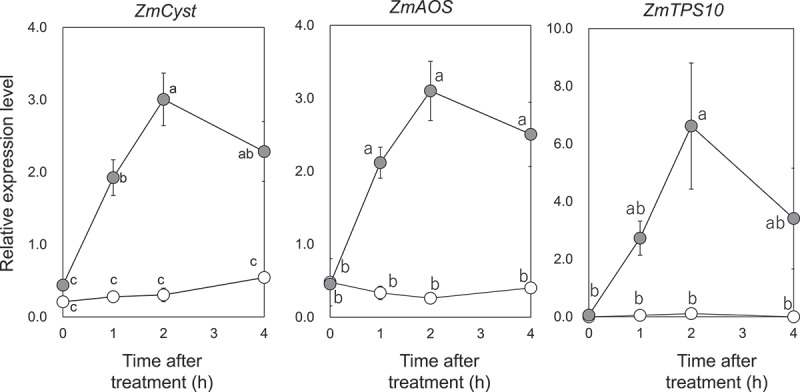
Time course of accumulation of transcripts of *ZmCyst*, *ZmAOS*, or *ZmTPS10* after spraying (*Z*)-3-hexen-1-yl acetate (**1**, Z3HAC) on the leaves of maize seedlings. The leaves were treated with (closed circular) or without (open circular) Z3HAC (**1**), and RNA was extracted for RT-qPCR at a given time point. Values represent means ± standard error of the mean (SEM) (*n*=4). Different letters indicate significant difference (*P*<0.05, two-way ANOVA followed by Tukey-Kramer).

The emission of GLVs from maize leaves was estimated to be 0.3–385.5 ppb when calculated from the data in the literatures.^20,21^ Mathematical model simulations indicated that concentration of Z3HOL deposited in the cuticular wax reached 370 µM when a plant would be exposed to 1 ppb of Z3HOL (**2**) vapor.^18^ Because the concentration in cuticle is linearly proportional to the atmospheric concentration^18^, GLV concentrations in the cuticular layer were expected to be in the range of sub-millimolar to several tens of mM. We previously confirmed in a study using tobacco BY2 cells that treatment in solution with 1 mM of volatile compounds effectively induces gene expression, and that this chemical treatment procedure successfully led to the identification of a candidate protein that likely interacts with a volatile compound.^22^ Accordingly, we prepared a 1 mM aqueous solution of Z3HAC (**1**) to treat maize seedlings. We sprayed the solution onto the upper ground part of 2-week-old maize seedlings, after which the expression levels of *ZmCyst*, *ZmAOS*, and *ZmTPS10* were measured. For all three genes, the effect of Z3HAC (**1**)-spray became apparent after 1 h, and at 2 h after spraying, their expression levels were the highest. The expression levels decreased thereafter (Figure 2).

### Effect of polar groups

To examine the effect of oxygen-containing polar groups, compounds **1** (Z3HAC), **2** (Z3HOL), **3** (Z3HAL), **4**, and **5** (Figure 1) were used to treat maize seedlings. Compound **4** is similar in structure to Z3HAC (**1**), but its oxygen atom is ether-bonded and is not susceptible to hydrolysis. Compound **5** was originally designed and synthesized to gain preliminary insight into the design of a chemical probe for the identification of HAC-binding proteins. Compound **5** has an ester bond similar to Z3HAC (**1**), but with a six-carbon acyl chain instead of the two carbons in Z3HAC on the acid side.

**Figure 3.**
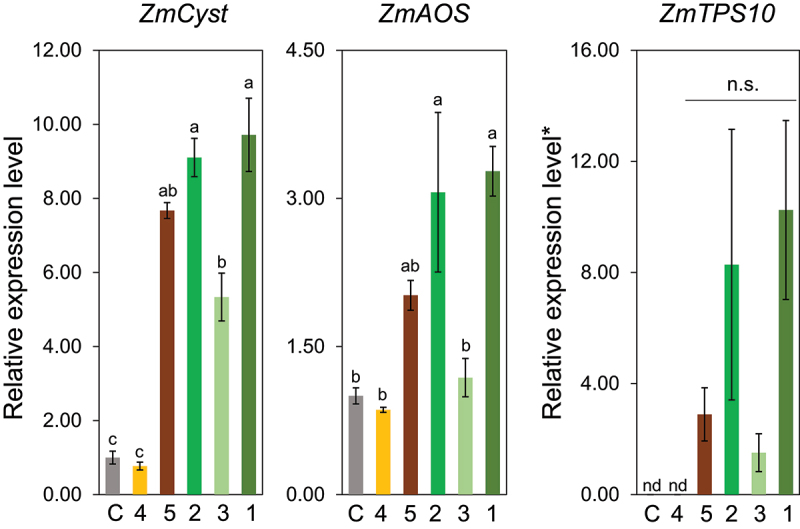
Effect of the structure of the polar group in compounds having (*Z*)-3-hexene backbone (compounds **1** to **5**) on the expression levels of *ZmCyst*, *ZmAOS*, and *ZmTPS10*. Values represent means ± SEM (*n*=4). Different letters indicate significant difference (*P<*0.05, one-way ANOVA followed by Tukey’s test).

Compound **2** (Z3HOL) induced the expression of *ZmCyst*, *ZmAOS*, and *ZmTPS10*, with abilities indistinguishable from those of Z3HAC (**1**) (Figure 3). Compound **3** (Z3HAL) significantly induced the expression of *ZmCyst* but almost failed to induce *ZmAOS*. Converting the ester bond of Z3HAC into an ether bond, as in compound **4** resulted in the total elimination of the inducing ability. In contrast, the ester compound **5**, which has the same structure on the alcohol side as Z3HAC, showed an almost equivalent ability to induce these three genes.

### Effect of the double bond

**Figure 4.**
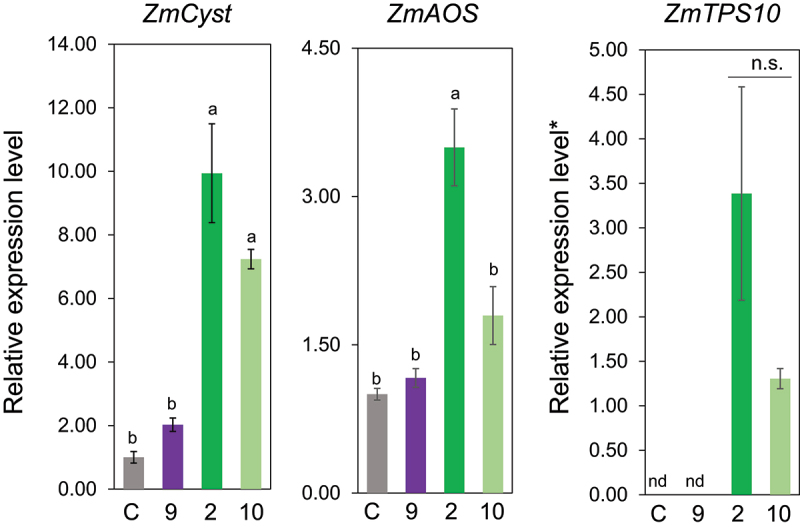
Effect of occurrence and geometry of a double bond in six carbon aliphatic alcohols (compounds **9**, **2**, and **10**) on the expression levels of *ZmCyst*, *ZmAOS*, and *ZmTPS10*. Values represent means ± SEM (*n*=4). Different letters indicate significant difference (*P<*0.05, one-way ANOVA followed by Tukey’s test).

Because no remarkable difference was detected in the gene induction ability, whether the polar group was alcohol or acetate, we decided to proceed with the structure-activity relationship analysis based on alcohols, for which various analogues of Z3HOL are available. Compound **10**, which has a double bond at the same position as Z3HOL (**2**), but in a different geometry (*E*), showed almost the same gene-inducing profile as that of Z3HOL (**2**) (Figure 4). In contrast, the removal of the double bond as in compound **9** almost completely abolished the ability to induce gene expression.

### Effect of the methylene length

**Figure 5.**
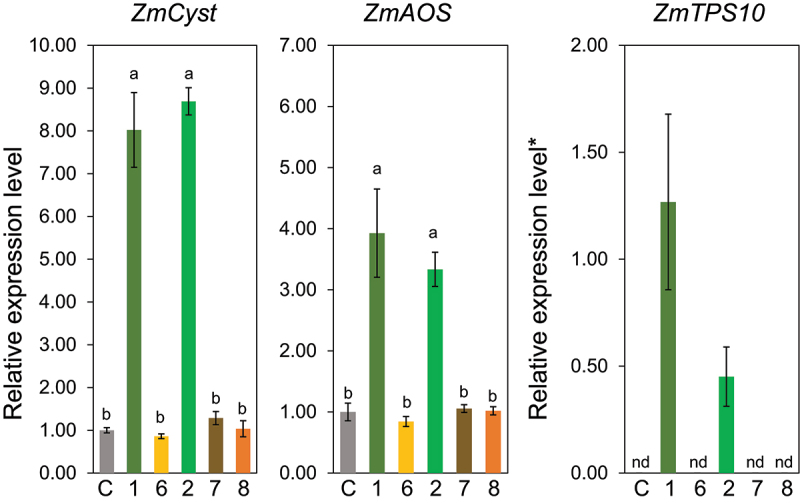
Effect of alkyl chain length at the polar group-side in (*Z*)-ω3-alken-1-ol (compounds **6**, **2**, **7**, and **8**) on the expression levels of *ZmCyst*, *ZmAOS*, and *ZmTPS10*. Values represent means ± SEM (*n*=4). Different letters indicate significant difference (*P<*0.05, one-way ANOVA followed by Tukey’s test).

**Figure 6.**
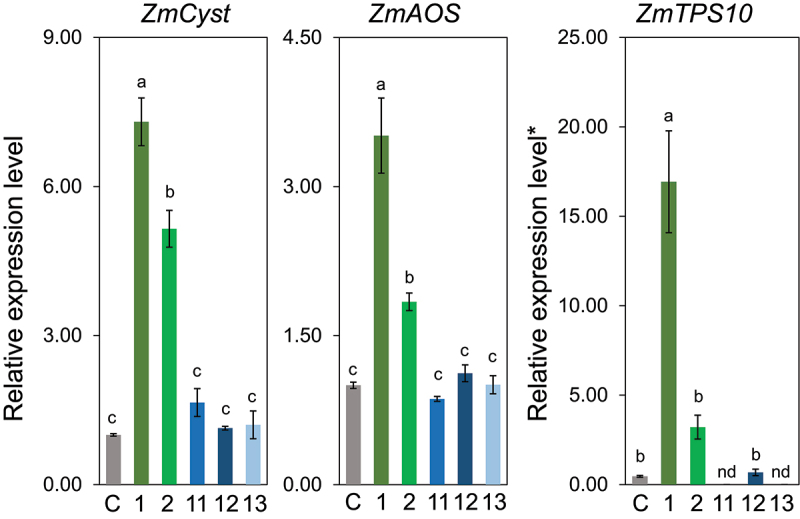
Effect of alkyl chain length at the ω-side in (*Z*)-3-alken-1-ol (compounds **2**, **11**, **12**, and **13**) on the expression levels of *ZmCyst*, *ZmAOS*, and *ZmTPS10*. Values represent means ± SEM (*n*=4). Different letters indicate significant difference (*P<*0.05, one-way ANOVA followed by Fisher’s LSD).

Next, we examined the effect of the aliphatic chain length between the polar oxygen and the double bond. Among the ω3-(*Z*)-alken-1-ol with different methylene lengths from the hydroxyl group to the double bond, that is, **2**, **6**, **7**, and **8**, only Z3HOL (**2**) showed prominent gene-inducing activity, but either a shorter or longer chain length than that of **2** eliminated the ability to induce the three genes monitored in this study (Figure 5). Such narrow specificity in terms of alkyl chain length was also evident in the alkyl chain length on the ω-side of (*Z*)-3-alken-1-ol; only Z3HOL (**3**) showed inducing activity, whereas neither (*Z*)-3-hepten-1-ol (**11**), (*Z*)-3-octen-1-ol (**12**), nor (*Z*)-3-nonen-1-ol (**13**) showed inducing activity (Figure 6).

### Treatment in gaseous form

**Figure 7.**
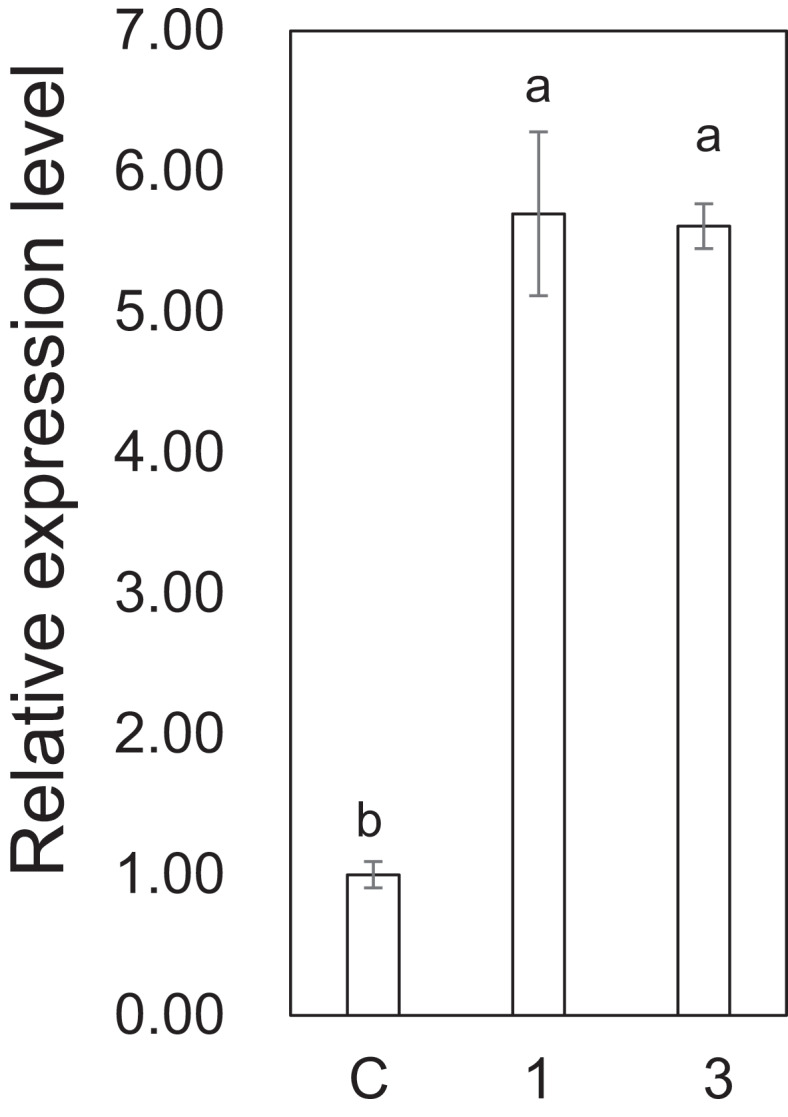
The relative expression level of *ZmCyst* after exposing maize seedlings to gaseous Z3HAC (**1**) and Z3HOL (**2**). Values represent means ± SEM (*n*=4). Different letters indicate significant difference (*P<*0.05, one-way ANOVA followed by Fisher’s LSD).

The ability of Z3HOL (**2**) to induce *ZmCyst* expression was examined in its gaseous form using an airflow system equipped with a microdispenser containing each compound dissolved in triethyl citrate (Figure S5). Under the experimental conditions employed here, 62.3 and 80.1 ng h^−1^ of Z3HAC (**1**) and Z3HOL (**2**) were detected at the outlet of the air-flow system. The airflows at the inlet and outlet were set to 0.3 and 0.2 L min^−1^. Accordingly, concentrations of Z3HAC (**1**) and Z3HOL (**2**) to which the maize seedlings were exposed were 5.19 and 6.68 ng L^−1^, i.e., 0.82 and 1.50 ppbV, respectively. These concentrations are equivalent to or even lower than those observed in the airflow from caterpillar-infested corn plants or cut leaf materials.^20,21,23^ Z3HOL (**2**) showed almost the same ability to induce *ZmCyst* as that of Z3HAC (**1**), even when employed in the gaseous form (Figure 7).

## Discussion

Plant volatiles mediate intraspecific and interspecific plant-plant communications, and GLVs, among others, are the volatiles that induce defense in receiver plants.^2^ With one GLV, (*Z*)-3-hexenol (**2**, Z3HOL), intake and glycosylation by the receiver plants account for the induced defense.^8^, but each component of the GLVs also induces the transcription of a subset of defense-related genes.^3,4^ In the latter case, GLVs are likely to be recognized by a factor and subsequently activate a signaling pathway, leading to the upregulation of defense-related genes. The major unresolved questions are which factor is responsible for the perception of GLVs, and how this factor induces the expression of a subset of defense-related genes. Several laboratories have independently reported the direct induction and/or priming of genes following GLV treatment in maize plants.^17,21,24,25^ Maize seedlings and GLVs appear to be suitable models to address this unresolved question. One approach to identify the factors involved in the perception of GLVs is structure-activity relationship analysis to confirm the essential structure to exert the activity. In this study, we conducted a structure-activity relationship analysis using (*Z*)-3-hexen-1-yl acetate (**1**, Z3HAC) as a lead compound by monitoring its ability to induce representative genes in maize seedlings.

When the ability of the compounds to induce *ZmCyst*, *ZmAOS*, and *ZmTPS10* was monitored, it was suggested that 3-hexen-1-ol is an essential structure for the expression of this ability. One of the key findings supporting this conclusion is that changing the ester moiety of Z3HAC (**1**) to an ether moiety resulted in a complete loss of its gene induction ability. In contrast, the inducibility did not substantially change when the acyl moiety of the ester was remarkably changed or when the ester was converted to the corresponding alcohol by removing the acyl moiety. Each component of the GLV family can interconvert in plant tissues.^5,6,15,26^ When Arabidopsis plants were exposed to Z3HAC (**1**), almost all the esters were hydrolyzed by the carboxyesterase AtCXE12 to Z3HOL (**2**) within 30 min.^26^ The involvement of carboxyesterase in the conversion of Z3HAC (**1**) to Z3HOL (**2**) in tomato fruits is also known.^27^ High carboxyesterase activity has also been reported in maize leaves even though its activity against Z3HAC (**1**) has not yet been examined.^28^ Structure-activity relationship analysis by monitoring the increased secretion of extrafloral nectar in lima beans, which is typically induced by the exposure of plants to Z3HAC (**1**), showed that the structure of the acyl moiety of the ester hardly affected its ability.^29^ The results shown here indicate that Z3HAL (**3**) showed a similar gene induction ability as those of Z3HAC (**1**) and Z3HOL (**2**), although the ability was somewhat reduced. It is also known that Z3HAL (**3**) can be reduced to Z3HOL (**2**) when intact Arabidopsis leaves are exposed to the vapor of Z3HAL (**3**).^5,15^ Thus, it can be concluded that Z3HOL (**2**) or compounds that can be converted to Z3HOL (**2**) can induce gene expression. To confirm this hypothesis, it was essential to elucidate the metabolic fate of Z3HAC (**1**) and Z3HAL (**3**) used to treat maize leaves.

Another prominent finding of this study is that the essential structure to evoke a defense response in receiver maize plants must be six-carbon alcohols with a double bond at position 3. The geometry of the double bond is not important because E3HOL (**10**) showed an almost equivalent ability to induce genes as that of Z3HOL (**2**). However, the indispensable function of a double bond was noticeable because *n*-hexan-1-ol (**9**) showed no gene inducing activity, whereas the overall spatial structure of *n*-hexan-1-ol (**9**) was almost the same as that of E3HOL (**10**). In 3-hexen-1-ol, the methylene length at either the α- or ω-terminal side of the double bond is strictly limited, and only 3-hexen-1-ol can exhibit this ability. Such narrow structural specificity has been demonstrated in maize plants by evaluating the increase in the amount of jasmonic acid.^30^ The formation of linalool and methyl salicylate in maize seedlings was induced by Z3HOL (**2**), but neither the compound without a double bond, *n*-hexan-1-ol (**9**), nor the one with one carbon longer methylene length at the ω-terminus, (*Z*)-3-hepten-1-ol (**11**), induced formation.^24^ Taken together, 3-hexen-1-ol is an essential structure to be appropriately perceived, and maize plants recognize not only the spatial structure but also the distribution of electrons in the structure.

Several possible mechanisms by which plants perceive GLVs have been suggested. GLVs with an unsaturated carbonyl moiety, such as E2HAL, are reactive electrophiles that deplete cellular reducing reagents, such as glutathione, resulting in changes in the cellular redox status that subsequently lead to plant stress responses.^14^ In this case, the functional group, i.e., α,β-unsaturated carbonyl group, is exclusively accountable to the plant response; therefore, the overall structure of the volatile compound is of little significance. Moreover, the penetration of volatile chemicals into biological membranes alters their physical properties, leading to stress responses in plants.^31^ If this is correct, plant responsiveness is expected to correlate with the logP value (oil/water participation coefficient) of the respective volatile compounds. However, as observed in this study, the response of maize plants did not clearly correlate with the hydrophobicity of the compounds used. This result is in agreement with the results of studies on the GLV response in lima bean using extrafloral nectar secretion as an indicator.^29^

Non-specific lipid transfer proteins (nsLTPs) in the cell wall are involved in VOC emission from inside the cell to the atmosphere.^32^ Considering that VOC uptake is the reverse process of VOC emission, it is possible that nsLTPs determine the efficiency of VOC uptake; in this case, the binding specificity of nsLTPs can determine the structural specificity of the plant response. However, mathematical models suggest that VOC uptake from the atmosphere into the cell does not necessarily require nsLTP capture and concentration.^32^ Because carboxyesterases are localized in the cytoplasm of plant cells, Z3HAC (**1**) probably enters the cell through the cuticle, cell wall, and cell membrane, and is hydrolyzed in the cytoplasm to the active substance, Z3HOL (**2**). Therefore, it is assumed that the tight structural specificity of GLV perception is largely determined by a factor in the cytoplasm. Glycosylation is one of the fates of Z3HOL (**2**) in cells. There are no known cases in which a specific volatile glycoside activates a signaling pathway that leads to gene induction in plants. It is also unknown whether the depletion of UDP-sugar, which would be consumed during the glycosylation process, activates the signaling pathway. In fact, at least in Arabidopsis, it has been reported that glycosylation occurs by incorporating structurally diverse alcohols, and the glycosylation process itself is not highly structure-specific.^33^. Accordingly, direct involvement of glycosylation in GLV perception can be excluded. Once Z3HOL (**2**) reaches the cell, it interacts in a structure-specific manner with an unknown factor to trigger another process, thereby activating a signal transduction pathway and inducing gene expression.

One of the objectives of our structure-activity relationship analysis was to identify the structural part that could be used to introduce a diazirine moiety for photoaffinity labeling and an acetylene moiety to isolate the factor by using click chemistry.^34^ Compound **5** was synthesized for this purpose because the acid moiety of the ester could be further modified to have a photolinker. However, the overall structure of Z3HOL (**2**) was found to be essential for gene induction monitored in this study, and the acyl moiety of the ester compound seemed to be removed by a carboxyesterase before it was perceived by the factor. Thus, there was no structural leeway for the introduction of functional groups. In the future, it will be necessary to employ other approaches, such as a forward genetic approach, rather than a chemical probe approach, to identify the factors that interact with Z3HOL (**2**). The effects of the abiotic environmental factors surrounding the plant, such as light quality, light intensity, humidity, and temperature, on the responses of plants against volatile compounds should also be carefully evaluated in future.

## Supplementary Material

Supplemental Material
