# Supplementary material for: Structure-activity relationship of volatile compounds that induce defense-related genes in maize seedlings
Source: Plant Signal Behav. 2023 Jul 16;18(1):2234115. doi: 10.1080/15592324.2023.2234115 (PMC10730182; doi:10.1080/15592324.2023.2234115)
Supplement: Supplemental Material [file KPSB_A_2234115_SM3172.docx]

Supplemental Table S1. Primers used for quantitative PCR analyses.

| Name | Direction | Sequence (5' -->3') | Remarks | Gene ID |
| --- | --- | --- | --- | --- |
| Cysteine proteinase  inhibitor 8 [*Zea mays*] | F | GGA CAT GAG CTG GCG ATT TT | *ZmCyst* | 103652885 |
|  | R | CAA GGA GCA CAA CAG GCA GA |  |  |
| Allene-oxide synthase1 [*Zea mays*] | F | GCG CGT CGT TGG ATA TAT GAT G | *ZmAOS* | 542150 |
|  | R | AGC GAC AAA CAC CTC CAA TC |  |  |
| Terpene synthase10  [*Zea mays*] | F | TGT GTC CAC GGT CCA ATG TT | *ZmTPS10* | 732751 |
|  | R | GTC CGC TGT CCT TGC AAA AT |  |  |
| *Zea mays* adenine phosphoribosyl transferase (apt1) | F | AGG CGT TCC GTG ACA CAA GA | *ZmAPT1* | 542148 |
|  | R | CTG GCA ACT TCT TCG GCT TCC |  |  |
